# Automated Construction of a Photocatalysis Dataset for Water-Splitting Applications

**DOI:** 10.1038/s41597-023-02511-6

**Published:** 2023-09-22

**Authors:** Taketomo Isazawa, Jacqueline M. Cole

**Affiliations:** 1 Cavendish Laboratory, Department of Physics, University of Cambridge, J. J. Thomson Avenue, Cambridge, CB3 0HE UK; 2 ISIS Neutron and Muon Source, STFC Rutherford Appleton Laboratory, Harwell Science and Innovation Campus, Didcot, Oxfordshire OX11 0QX UK

**Keywords:** Photocatalysis, Catalysis

## Abstract

We present an automatically generated dataset of 15,755 records that were extracted from 47,357 papers. These records contain water-splitting activity in the presence of certain photocatalysts, along with additional information about the chemical reaction conditions under which this activity was recorded. These conditions include any co-catalysts and additives that were present during water splitting, the length of time for which the photocatalytic experiment was conducted, and the type of light source used, including its wavelength. Despite the text extraction of such a wide range of chemical reaction attributes, the dataset afforded good precision (71.2%) and recall (36.3%). These figures-of-merit were calculated based on a random sample of open-access papers from the corpus. Mining such a complex set of attributes required the development of novel techniques in knowledge extraction and interdependency resolution, leveraging inter- and intra-sentence relations, which are also described in this paper. We present a new version (version 2.2) of the chemistry-aware text-mining toolkit ChemDataExtractor, in which these new techniques are included.

## Background & Summary

Photocatalytic water splitting is an active field of research since the production of hydrogen from sunlight and water offers a promising solution for environmentally friendly energy. As a result, research into this field has exploded in recent years and there is an interest in applying machine learning to this field^1^. Yet, it can remain difficult to model and predict novel photocatalysts. Past efforts^2,3^ have focussed primarily on electronic-structure calculations that employ density-functional theory (DFT), building off datasets that contain a large number of DFT results such as the Materials Project dataset^4^.

This study takes a different approach by developing a dataset containing experimental data that includes commonly reported key photocatalytic reaction attributes and their associated catalytic-efficiency metrics. These metrics were chosen from the academic literature, and include the solar-to-hydrogen efficiency (STH), the apparent quantum yield (AQY), and the amount of hydrogen emitted per unit of time or per unit amount of photocatalyst^5^. However, these catalytic-efficiency metrics alone are often not enough to discern the effectiveness of a photocatalyst given that catalytic activity can vary wildly based on the chemical reaction conditions under which the water-splitting reaction takes place. These conditions can include the light source used, the chemical additives that may be present, and even the photoreactor configuration^6,7^. To take these environmental conditions into account, the dataset introduced in this paper also includes the co-catalyst, any chemical additives that are present in the mixture, the length of time for which the experiment was conducted, and the type of light source used, including its wavelength.

The extraction of such a large number of chemical reaction attributes presents difficulties for text mining. Firstly, multiple catalytic activities for multiple compounds are often listed in a single document, or at times, even in a single sentence, complicating the association between photocatalysts and the water-splitting activities in their presence. Secondly, the photocatalytic activity and the associated environmental conditions during water splitting are often presented across multiple sentences.

As an example of these challenges, consider the following passage, where the relevant data that we wish to extract and appropriately associate are highlighted. “*Therefore*, *all HOCN samples exhibit enhanced photocatalytic H*_2_ *evolution rates under****visible light irradiation (λ*** > ***420*** ***nm)***. *In particular*, ***HOCN***_***4***_ *produced a remarkable enhanced photocatalytic H*_2_ *production rate of****1140*** ***μmol h***^−1^***g***^−1^ *under visible light irradiation*, *which is much higher than that of pure****g-C***_**3**_***N***_4_ (CN, ***82*** ***μmol h***^−1^***g***^−1^***)****. Further*, ***HOCN***_***4***_ *exhibits a high H*_2_ *production rate of* ***477 and 91*** ***μmol h***^−1^***g***^−1^ *under light illumination with longer wavelengths (i.e*., *λ* > ***500*** ***nm and λ*** > ***800*** ***nm***, *respectively) […] A****300*** ***W xenon lamp (CEL-HXF300***, ***perfectlight***, ***Beijing)**** was used as the light source*.”^8^.

This paper presents novel techniques for the knowledge extraction of these distributed photocatalytic data and new interdependency-resolution techniques that allow the data to be appropriately associated during the text-mining process. Application of these newly developed techniques on a corpus of 47,357 papers resulted in a photocatalysis dataset with 15,755 records. Checking the validity of these data records using a subset of these papers revealed a precision of 71.2% and a recall of 36.3%. ChemDataExtractor has been made available at https://github.com/CambridgeMolecularEngineering/chemdataextractor2, and the the specific version and additional code used for this paper have been made available on Figshare^9^. The Figshare also contains the data extracted in both normalised and denormalised JSON formats, and they have also been converted to a CSV format, as well as the hand-annotated test dataset and a separate hand-annotated dataset used during development in the same formats. These can be used for the purpose of photocatalysis property prediction or for evaluating any new data-extraction methods in this domain of chemistry.

## Methods

### Acquiring a corpus of data sources

The corpus was gathered using the web-scraping capabilities included with ChemDataExtractor. Two search queries, “photocatalytic water splitting activity” and “photocatalyst hydrogen water activity”, were used to scrape articles published by the Royal Society of Chemistry and Elsevier, with the permission of the publishers. These scraped papers were then filtered based on whether the full-text articles were available in structured HTML/XML formats. This procedure resulted in a total of 47,357 papers being gathered for this work, consisting of 35,753 papers published by Elsevier and 11,604 published by the Royal Society of Chemistry.

### Knowledge representation for photocatalysts

**Fig. 1:**
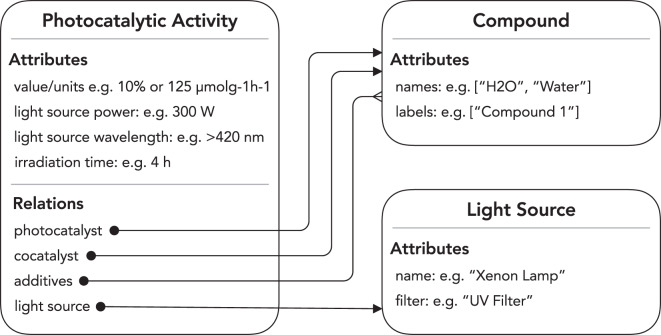
The knowledge representation selected for photocatalytic activity.

The highly variable nature of photocatalytic water-splitting activity depending on the chemical reaction conditions^6,7^ necessitated the construction of a complex knowledge representation for this study. This was a good match for the highly flexible knowledge-representation capabilities of the text-mining toolkit ChemDataExtractor version 2, which allows data extraction of nested models where there are complex relations between properties^10^. The model constructed within this framework contained the photocatalytic activity itself, as well as the experimental conditions under which the photocatalytic reaction took place, as illustrated in Fig. 1.

We chose three different measurement metrics to describe for photocatalytic activity: the solar-to-hydrogen efficiency (STH), the apparent quantum yield (AQY), and the amount of hydrogen emitted per unit of time or per unit amount of photocatalyst present in the chemical reaction. The first two measures are expressed as percentages, while the amount of hydrogen emitted is expressed differently depending on the paper, with some of them expressing this metric^11^ in units of *μ*mol g^−1^, others^12^ as *μ*mol h^−1^, and some others^13^ as *μ*mol g^−1^ h^−1^. With regards to the environmental conditions, we chose those that seemed to be most commonly reported and most influential on the catalytic activity, while being feasible to extract and represent; namely, the wavelength of the light source, the type of light source, the power of the light source, co-catalysts used, and any chemical additives used, such as sacrificial reagents. While influential to the photocatalytic activity, some conditions, such as the reactor configuration, were not extracted in this work due to a lack of suitable quantitative knowledge representation that would be useful in downstream tasks, and others, such as the relative amount of co-catalyst, were omitted due to the diverse ways in which they could be represented.

### Knowledge extraction

While the complex nature of this knowledge representation was necessary in order to reflect the number of variables that affect photocatalytic activity, this resulted in a variety of challenges. Considering again the example passage given above, there are two key features that make the extraction of its photocatalysis-related information difficult. Firstly, there are numerous pieces of photocatalysis-relevant information within each sentence. It is particularly challenging to extract information from the second sentence (*Further*, *HOCN4 exhibits a high H2 production rate of 477 and 91* *μmol h*^−1^*g*^−1^ *under light illumination with longer wavelengths…*) as a successful system would need to not only extract data from multiple parts of the sentence but also infer the association between data.

Secondly, photocatalysis-relevant information can be written across multiple sentences or multiple paragraphs. This poses challenges for interdependency resolution, since the merging of information based on text proximity alone can result in false positives.

**Fig. 2:**
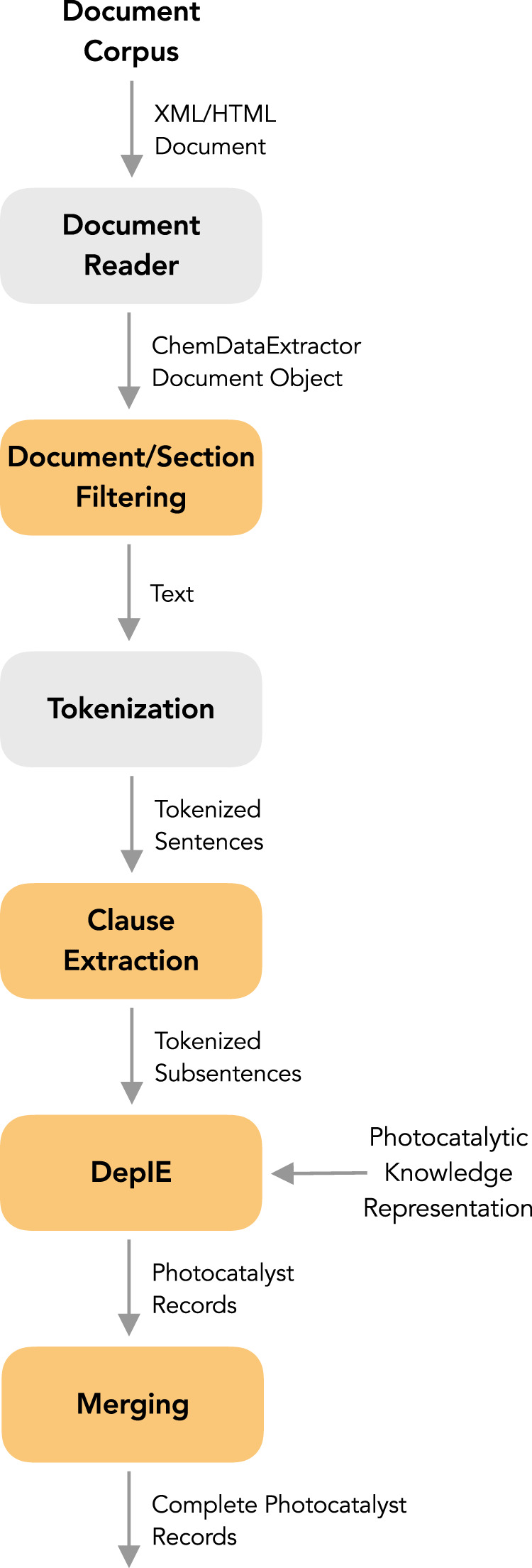
The overall pipeline used to extract photocatalysis records. Steps that were newly developed or enhanced for this work are highlighted in yellow.

We present a number of novel techniques that were designed and implemented in order to overcome these challenges. The corresponding overall data-extraction pipeline is presented in Fig. 2.

#### Clause extraction

To facilitate the process of correctly associating data within a sentence, we first run a “clause extraction” process, where we attempt to break down larger, more complex sentences that include coordinating conjunctions into sets of basic clauses. A simple example of this would be the extraction of clauses *“A has quality α*” and *“A has quality β*” from the sentence “*A has quality α and quality β*”. The motivation behind this method is that the creation of these more basic sentences should then be easier for any downstream information-extraction system to understand. The identification of the conjuncts in the original sentence is known as coordination resolution, and this process of clause extraction adds the additional step of constructing the appropriate clauses given these conjuncts. Before describing our algorithm for this task, consider how a human would approach this problem.

In this simple example, we are taking one sentence and splitting it into two clauses when we encounter a coordination. We define this operation, where the number of sentences is multiplied by the number of conjuncts, as a *“multiplication*” in clause space.

Next, consider the slightly more complicated example sentence, *“A and B have quality α and β respectively”*. Here, as with the previous sentence, we initially derive *“multiply”* the sentence at the first phrase, *“A and B”*, so that we end up with two clauses: *“A have quality α and β respectively”* and *“B have quality α and β respectively”*. When we arrive at the next coordination, *“α and β”*, we know that A is tied with *α* and B is tied with *β* respectively, so we would apply a different operation, an *“adding”* in clause space, which results in *“A have quality α respectively”* and *“B have quality β respectively”*. While this does not result in perfect English, it does result in sentences which are much easier for a computer to understand via simple parsers.

While these are simple example sentences, with simple operations, these explain how we, as humans, approach this task. Under this framework, the problem of clause extraction can then be reduced to a problem consisting of two parts: the identification of conjuncts, i.e. coordination resolution, and the selection of operations that associate them.

Language models have been used to perform this task of identifying conjuncts (e.g. A, B, *α*, *β* in the above examples, although these conjuncts can be multiple words in practice) by evaluating the probabilities of sentences where each potential conjunct is used, and picking the conjunct that yields the highest probability^14^.

We instead take advantage of the recently improved precision of syntactic dependency parsing using neural methods (>90% precision on the Penn Treebank dataset^15^) and take the simpler path of identifying the conjuncts by finding subtrees in the syntactic dependencies that are found for the sentence, originating at the words which are given the conjunct relation.

The operation used for each set of conjuncts is then determined using the following heuristics:

- If the number of conjuncts in a certain coordination is equal to the number of clauses generated prior to this coordination, we apply an *“addition”* operation.
- If the number of conjuncts in a certain coordination differs from the number of clauses generated prior to this coordination, we apply a *“multiplication”* operation, unless changing the previous operation from an *“addition”* to a *“multiplication”* allows these coordinations to be processed using an *“addition”*.

**Fig. 3:**
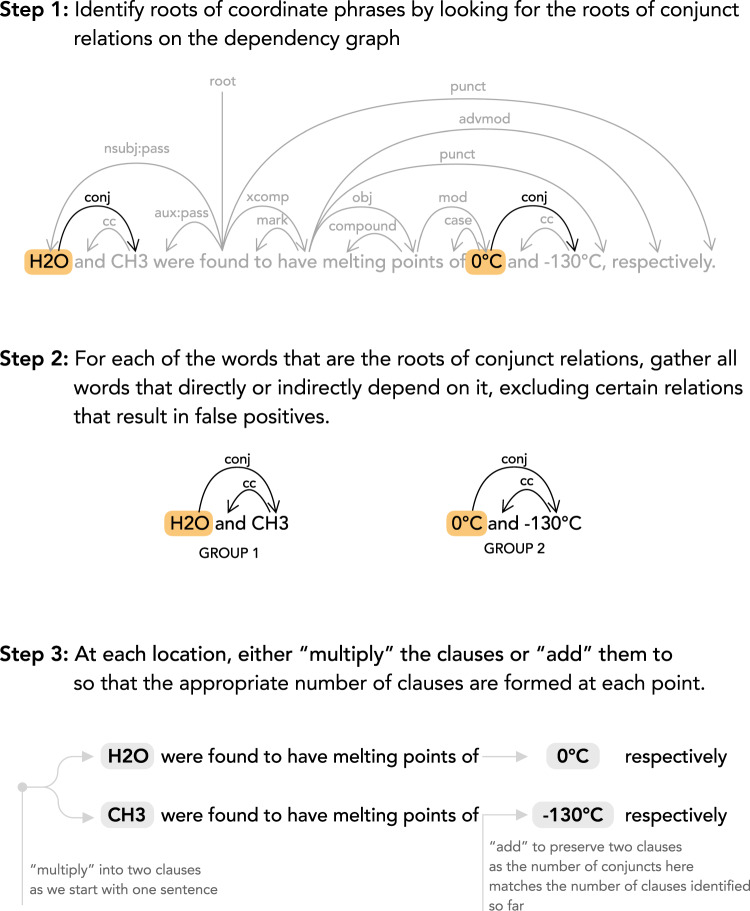
A step-by-step example of clause extraction. The types of dependencies (conj, cc, etc.) are according to the Universal Dependencies framework [18]. Of particular relevance is conj, shorthand for a conjunct, which is the relation between two words connected by a coordinating conjunction, e.g. *and*.

These heuristics, while simple, correctly resolve the examples shown above and even some more convoluted cases such as *“A and B tested under conditions 1 and 2 had qualities α*, *β*, *γ*, *δ”*. A step-by-step example of this, for a more realistic sentence can be seen in Fig. 3. This method works particularly well for the following sentence from one of the papers used to generate our photocatalytic-activity dataset: *“Deduction from OCP measurements was consistent with the measured water-splitting activities of particulate SrTiO3:Al/Pt*, *where the H2 evolution rates in H2 and O2 environments*, *respectively*, *were shown to be 13* *μmol h*^−1^ *and 0* *μmolh*^−1^, *respectively”*^12^, where the following two clauses are extracted by our implementation of this algorithm, using Stanza^16^ to provide the dependencies in the form of Universal Dependencies^17^: *“Deduction from OCP measurements was consistent with the measured water-splitting activities of particulate SrTiO3:Al/Pt*, *where the H2 evolution rates in H2 environments*, *respectively*, *were shown to be 13* *μmol h*^−1^*.”* and *“Deduction from OCP measurements was consistent with the measured water-splitting activities of particulate SrTiO3:Al/Pt*, *where the H2 evolution rates in O2 environments*, *respectively*, *were shown to 0* *μmol h*^−1^, *respectively.”*

#### Enhancing rule-based extraction with dependencies

With ChemDataExtractor 2^10^, simple rule-based parsers were automatically generated based on the knowledge representation, using the names and types of the parameters. However, while this worked well for tables owing to their semi-structured nature, this was prone to having either low recall or low precision when applied to text due to the simplicity of the generated parsers.

While clause extraction can be helpful, information extraction would still be difficult as even “basic” sentences with no coordinations can still contain multiple bits of information, and simpler automatically generated parsers can succeed at identifying the spans of text containing facts, but fail to correctly associate information. For example, take the following sentence: *“The binary promoted Cu(0)–N-TiO2 photocatalyst prepared from N-TiO2 by “one-pot” photodeposition of copper in the suspension showed a high activity in the in situ hydrogen generation under near-UV/visible light with glycerol in water*, *up to 675* *μmol g*^−1^ *h*^−1^*”*^18^. Here, we cannot use the previous algorithm to extract clauses, but there are many chemical entities (Cu(0)–N-TiO2, N-TiO2) that could be associated with the activity value (675 *μ*mol g^−1^ h^−1^).

**Fig. 4:**
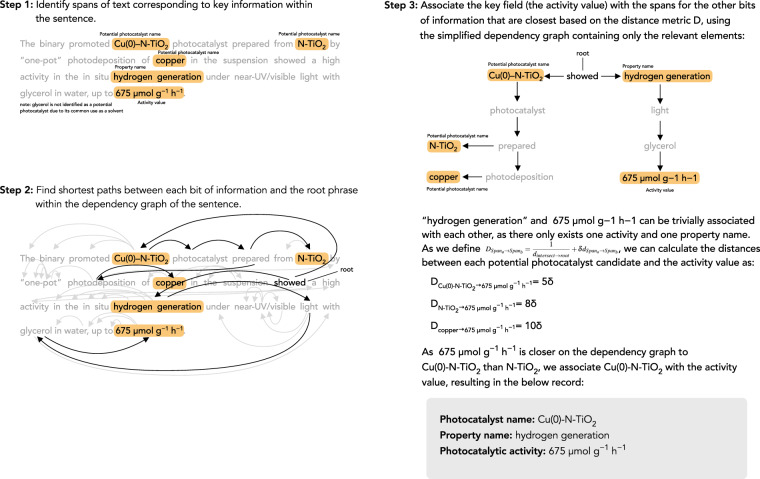
A step-by-step example of the DepIE algorithm. In practice, the additive (glycerol) and the light-source type (near-UV/visible light) are also associated using DepIE, but have been omitted from this example for the sake of simplicity. In practice, we get the dependency graph, including the root phrase, from Stanza^16^.

We introduce the Dependency-based Information Extraction (DepIE) algorithm, which aims to address this task of associating the correct spans with each other. An example of the application of the algorithm to the above sentence can be seen in Fig. 4.

The algorithm takes as input a “key field”. The key field is defined as the field within the knowledge representation for which the identified spans will be most accurate, and which are necessary for a record to exist in a sentence. In the case of photocatalytic activities, we use the activity value (i.e. 675 *μ*mol g^−1^ h^−1^ in the example above), as the “key value”.

The first step of the DepIE algorithm is to identify all spans that contain chemically relevant information in a sentence (e.g. 675 *μ*mol g^−1^ h^−1^ or Cu(0)–N-TiO2 in the example sentence above) and the syntactic dependency graph for the sentence. In practice, these spans of text that contain relevant information are either supplied by hand-crafted rules (e.g. for finding photocatalytic activity values), or via the named-entity-recognition capabilities of ChemDataExtractor^19^ (chemical-entity names). The syntactic dependency graph is supplied by Stanza^16^ in our implementation.

The second step of the DepIE algorithm is to calculate the “distances” between each span that corresponds to a key field and the spans that corresponds to each of the other fields. The distance *D* is defined as the following:

$${D}_{Spa{n}_{a}\to Spa{n}_{b}}=\frac{1}{{d}_{intersect\to root}}+\delta {d}_{Spa{n}_{a}\to Spa{n}_{b}}$$

where *d*_*a*→*b*_ is defined as the number of edges in the syntactic dependency graph between phrases, the root is the root phrase in the dependency parse, and *δ* is a small number so that the second term can be used as a tiebreaker. This metric for distance was chosen instead of just using the number of edges in the dependency graph between the spans because we follow the intuition that when two spans are siblings on the dependency graph and are far away from the root phrase, they should be more closely related than if they were siblings from the root phrase.

Once *D* has been calculated for each pair of key field spans and spans for other fields, we iterate through all of the key field spans and greedily assign the spans from other fields such that the distance is minimised.

The DepIE algorithm also associates a confidence with each association of spans, which is defined as

$$max\left\{\frac{1}{\frac{{D}_{min}}{{D}_{2ndsmallest}}+1},\frac{1}{{n}_{key}{n}_{other}}\right\}$$

where *D*_*min*_ is the minimum distance and *D*_2*ndsmallest*_ is the second smallest distance between key field spans and other field spans. *n*_*key*_ and *n*_*other*_ are the numbers of key field spans and other field spans, respectively (e.g. for the above example, *n*_*key*_ is 1, as there is only one activity value, while *n*_*other*_ is 2 for the chemical name). The second term is the probability of associating the correct spans with a random guess, while the first term is a rough approximation for the probability of associating the correct spans using DepIE. The first term is based on the intuition that if *D*_*min*_ was equal to *D*_2*ndsmallest*_, then we would be effectively randomly guessing, while if *D*_*min*_ much smaller than *D*_2*ndsmallest*_, we would expect the confidence to be close to unity. We then take the maximum between the two terms, with the assumption that DepIE’s guess will be better than a random guess. This confidence estimate is used to help prioritise merging of better quality extracted information, as detailed in the next section.

The novelty of this algorithm lies in its generalisability to the association of different user-specified properties by composing arbitrary recognisers for each of the fields, which can range from simple ones for property names to more complex ones for named-entity recognition. This permits this approach to be used not only to extract photocatalytic-activity values, but also to recognise the correct additives or co-catalysts. This open-endedness lies in comparison to approaches such as RelEx^20^ that use more specific relations, limiting generalisability, or Open Information Extraction systems such as ClauseIE^21^, that do not focus on the Knowledge Base Construction of specific properties based on a user-defined schema as we do here.

#### Leveraging document structure and confidences for merging

While clause extraction and DepIE can extract knowledge well from individual sentences, in the case of photocatalytic water splitting, owing to the number of reaction attributes involved, it is not often the case that all pertinent information will be contained in one sentence. For this reason, we need to merge information from across different sentences.

Intuitively, using text proximity as a heuristic for prioritising merging seems to be a good idea, and ChemDataExtractor has always exploited this^22^. In this work, we extend this heuristic in three ways.

First, we allow limits to be imposed on the ranges of text across which information can be merged, as it is not so likely that related facts be mentioned at opposing ends of a paper, although it does happen. Take, for example, the case of experimental method and results sections. These may be very far apart spatially in a paper, but the information contained in one section is highly likely to be pertinent to the other. To account for this eventuality, we allow the manual definition of closely related sections and treat them as though they are neighbouring sections, thereby allowing relevant information to be merged in even when the sections are far away in the document.

Finally, we take into account the quality of the extracted information when merging data (e.g. associating the light source used and the photocatalytic activity) by ordering candidate data for merging using a score that is defined as follows to better surface high-quality records:

$$\frac{spatial\;distance\;inbetween\;extracted\;information\;in\;document}{confidence\;of\;record},$$

where the confidence of a record is calculated using the confidence ascribed to extraction by DepIE.

#### Filtering content

Review papers are much longer than regular research articles, and their content consists of a survey of key results from many such articles. They therefore contain data about many independent photocatalytic reactions, which are scattered throughout the paper. Review papers thus pose difficulties in our information-extraction pipeline. For example, reaction conditions from different experiments would be merged where they should not be. The use of review papers in data extraction would also have a disproportionate effect on the overall precision of the resulting dataset, because not only is it hard to extract the correct data from them, they also contain many records to extract incorrectly. Review papers were therefore filtered out from our data-extraction process, and we extracted data from only regular papers, which tended to exclusively describe one photocatalytic chemical reaction. For the same reason, certain journals (e.g. the Neuron journal, or those from Cell Press), which are either off topic or seem to encourage the reporting of many results about multiple chemical reactions, were also filtered out.

Furthermore, a labelled dataset, that was manually created for validating the pipeline during development, showed that the results of scraping involved many papers where photocatalysis was only mentioned in passing. Such papers that simply mention the topic of photocatalysis would harm precision greatly as many of the sentences contained within them would, on their own, be indistinguishable, even for a human, from a sentence that would be in an “in-topic” paper such that it would produce incorrect records. An example of such a sentence is *“We obtained the utmost hydrogen evolution i.e*., *10200* *μmol h*^−1^ *g*^−1^ *for the naked ZAS (without a co-catalyst) catalyst under visible light*, *which is much higher than the earlier reported photocatalysts.”*^23^ This sentence describes the photodecomposition of H2S, but on its own, it is indistinguishable from a sentence about photocatalytic water splitting.

Although topic modelling^24^ would enable the distinction of such types of text, such modelling would require a large corpus of papers. Instead, we found that filtering based on the title, using simple block/allow lists, was enough to remove any “out-of-topic” papers in the validation dataset, and this also generalised well to the test dataset.

Finally, certain sections of some papers were also prone to contain disproportionately more false positive records than true positives. For example, many papers contain sections on characterising or synthesising the photocatalysts, which confuse the merging step in the information-extraction process since these would also often mention light sources or reaction times. A filter was implemented to address the issue, whereby sections or sentences which seemed irrelevant to the topic would be skipped. The sections that would be skipped were also chosen manually based on the development dataset, and this generalised well to the test dataset.

#### Tuning parameters

While the DepIE parsers were generated from the knowledge representation in this data-extraction process, certain parts of the pipeline had to be hand tuned, based on findings from use on a small development dataset that comprises 30 randomly selected, hand-labelled papers, and these annotations have been made publicly available^9^.

The first thing that had to be tuned here was the filter, where the keywords used to identify relevant sections and documents were picked. Related sections and document-text range limits for merging that worked well for the corpus were also identified and added to the pipeline. Finally, the dimensionalities of the photocatalytic properties and their names, including their synonyms where relevant, were ascertained for the knowledge representation so that the automatically generated parsers could function well.

## Data Records

The hand-annotated data and extracted data are all available online on Figshare^9^, in both normalised and normalised JSON forms. The normalised form can be interpreted by any standard JSON reader, while the normalised form can be read using the CDEDatabase (https://github.com/ti250/cdedatabase) library to create Python objects where the user can access more features such as standardised values.

In either case, the filename contains the type of the record, e.g. *ApparentQuantumYield*, and the fields are as described in Fig. 1. When using the dataset, it is important to note that the compound names for the photocatalyst and co-catalyst have not been normalised, and are kept as written in the paper, meaning that they are often expressed in non-standard forms.

## Technical Validation

To evaluate the dataset produced, a random sample of 330 open-access papers was taken from the overall dataset, with 220 of these papers being published by Elsevier and 110 papers being published by the Royal Society of Chemistry. These papers were then annotated by hand to create a test dataset. We computed precision and recall both for exact matches and relaxed matches.

For an exact match, the extracted value must precisely match the way that this was expressed in the original paper. This was particularly problematic in the case of chemical-entity names owing to the importance of structure and loading in photocatalysis. For example, an extraction of just “CDSe” would not be an exact match for “CDSe QDs” and similarly, “TiO2” would not be a match for “Ni(OH)2-decorated TiO2”. These partial matches are still useful extraction results which add only correct information, albeit incomplete information. To account for these cases of incomplete information, we also measure precision and recall with relaxed matches, and report these alongside each other. The results can be seen in Table 1.

**Table 1:** Precision and Recall on our corpus of 330 hand-labelled open-access papers.

	Relaxed Matches			Exact Matches		
	Precision	Recall	F1	Precision	Recall	F1
**Overall**	71.2	36.3	48.1	67.4	35.1	46.1
Activity value/units	91.8	47.2	62.3	91.8	47.2	62.3
Photocatalyst name	68.5	40.3	50.7	49.3	32.7	39.3
Co-catalyst name	51.2	48.8	50.0	48.8	47.6	48.2
Additive name	77.5	25.0	37.8	77.5	25.0	37.8
Irradiation time	61.8	38.2	47.2	61.8	38.2	47.2
Light source name	62.8	26.5	37.2	62.8	26.5	37.2
Light source filter	66.7	8.3	14.8	66.7	8.3	14.8
Light source wavelength	61.0	39.7	48.1	61.0	39.7	48.1

**Fig. 5:**
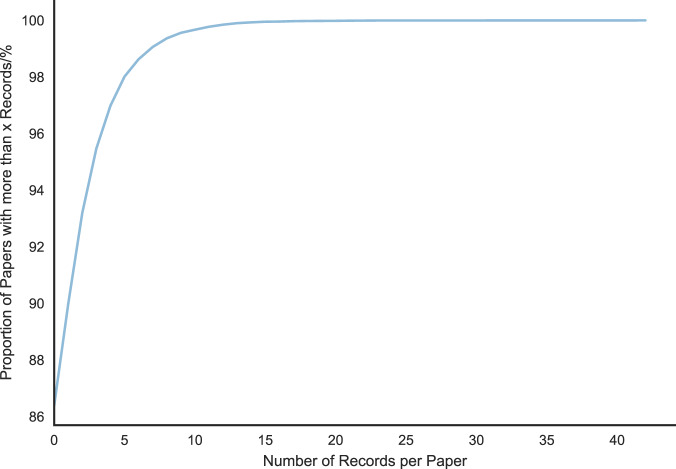
Cumulative distribution of number of records per paper in the extracted dataset. The maximum number of records in a single paper was 42, and despite the specific search query, 86% of papers contained no records. Also note that the majority of papers with records contained 5 records or fewer, which is to be expected as review papers were filtered out.

It was found that a large proportion of the papers in this test set actually did not contain any records. In fact, 90% of the papers in the test set did not have any records at all. As Fig. 5 shows, this was found to be the case with the full, automatically extracted, dataset as well, so this is not likely to be an issue with the test dataset but rather a *feature* of how the data are distributed within these papers. The high precision of 71.2%, despite this distribution, shows that our method effectively filters out false positives from these papers.

**Fig. 6:**
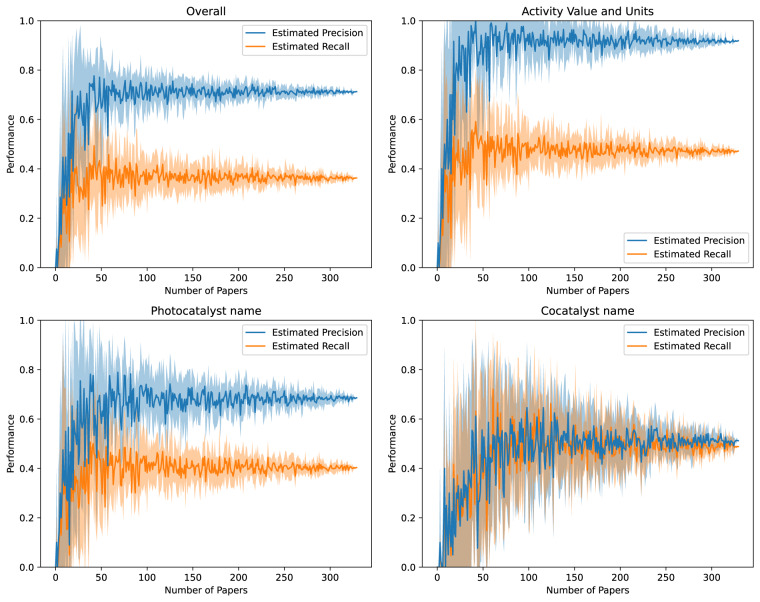
Convergence of performance metrics with the number of papers labelled in the test set. Due to the distribution of records per paper (as demonstrated in Fig. 5), the specific *n* papers selected to be labelled affected the performance in an outsized way, so the standard deviations for performances were estimated for labelling *n* papers by sampling *n* papers from the test data multiple times.

We also evaluated whether the dataset was large enough or not, by evaluating the convergence of the precision and recall metrics with the number of papers labelled, as shown in Fig. 6. Due to the distribution of records across papers, the specific papers that were being included in the *n* papers used to calculate the precision and recall would change the results; so we also calculated a standard deviation on the metrics based on 10 separate samples of *n* papers. While this gives a bad estimate as *n* approaches 330, due to all the samples containing the same papers, it nevertheless provides an additional illustration of whether or not the dataset size was big enough to accurately judge precision and recall.

Another thing to note in the results is that our system did not perform as well at extracting light-source filters and names, where the recall is much lower than with any other field (see Table 1). This anomaly occurs because, we chose, by hand, some common filter names from experience and from reading the development set of papers. This approach meant that while our system could precisely find light sources, our system failed to generalise well to “out-of-distribution” papers. This shortcoming was not observed for other types of data within our data set. This limitation, alongside the relatively low recall even for other fields (36.3% for relaxed matches overall) resulted in very few complete records being extracted, with many extracted data missing reaction conditions, in particular light-source information.

Despite its incompleteness, this extraction of reaction conditions is important to grasp because this study represents our first application of ‘chemistry-aware’ NLP to data that report along a time base, e.g. a chemical reaction. Our previous work has delivered large datasets that contain {chemical,property,value,unit} records, including those with multiple properties^25,26^. However, data that represent a chemical reaction include multiple chemical names, reagents, adducts, methods, products and various associated properties and related information that measure performance aspects of a reaction, e.g. its efficacy or activity. This already complicated set of data attributes is all the more complex when one considers that these data types exist at different points of time. This work therefore represents a major step forward and describes the state-of-the-art which is of course not perfect. Yet, its further development is needed if scientists are going to become able to properly tackle textual extraction of chemical-reaction data. We hope that our paper will offer insights that others can use to progress this area of data extraction for synthetic chemistry.

**Fig. 7:**
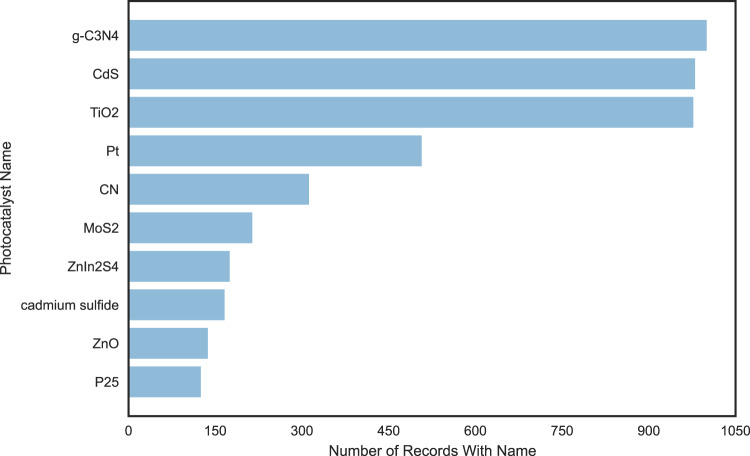
Top 10 catalysts in terms of number of reported results on photocatalytic water-splitting activity in our dataset.

As a common-sense check, the most-commonly occuring photocatalysts in the dataset were also analysed, yielding Fig. 7, with the results being in line with chemical expectations.

## Usage Notes

Both the extracted data and the hand-annotated data are available on Figshare^9^ and are provided in a denormalised JSON form which can be interpreted by any standard JSON library, or a normalised form which can be read with the CDEDatabase package (https://github.com/ti250/cdedatabase) to create Python objects. A converted CSV version of the data is also available in the respective repositories. Documentation and sample code to read either format is also provided on Figshare, alongside the scripts used to create the figures in this paper. The extracted dataset could be used for photocatalytic-material discovery and the hand-annotated dataset could be used for the evaluation of future efforts in chemical-information extraction pipelines to provide insights into the use of NLP to mine textual data from synthetic chemistry.

## Data Availability

ChemDataExtractor 2.2 is available at https://github.com/CambridgeMolecularEngineering/chemdataextractor2, and the automatically generated dependency parser, and the files used to specify the knowledge representation have been made open source and are available on Figshare^9^.
